# Simulation of the transition metal-based cumulative oxidative potential in East Asia and its emission sources in Japan

**DOI:** 10.1038/s41598-021-85894-z

**Published:** 2021-03-22

**Authors:** Mizuo Kajino, Hiroyuki Hagino, Yuji Fujitani, Tazuko Morikawa, Tetsuo Fukui, Kazunari Onishi, Tomoaki Okuda, Yasuhito Igarashi

**Affiliations:** 1 Meteorological Research Institute (MRI), Japan Meteorological Agency (JMA), Nagamine 1-1, Tsukuba, Ibaraki 305-0052 Japan; 2 Faculty of Life and Environmental Sciences, University of Tsukuba, Tsukuba, Ibaraki 305-8572 Japan; 3 Japan Automobile Research Institute (JARI), Tsukuba, Ibaraki 305-0822 Japan; 4 National Institute for Environmental Studies (NIES), Tsukuba, Ibaraki 305-8506 Japan; 5 Institute of Behavioral Sciences, Shinjuku, Tokyo 162-0845 Japan; 6 St. Luke’s International University, Chuo, Tokyo 104-0044 Japan; 7 Faculty of Science and Technology, Keio University, Yokohama, Kanagawa 223-8522 Japan; 8 Institute for Integrated Radiation and Nuclear Science (KURNS), Kyoto University, Kumatori, Osaka 590-0494 Japan; 9 College of Science, Ibaraki University, 2-1-1 Bunkyo, Mito, Ibaraki 310-8512 Japan

**Keywords:** Environmental impact, Atmospheric chemistry

## Abstract

The aerosol oxidative potential (OP) is considered to better represent the acute health hazards of aerosols than the mass concentration of fine particulate matter (PM_2.5_). The proposed major contributors to OP are water soluble transition metals and organic compounds, but the relative magnitudes of these compounds to the total OP are not yet fully understood. In this study, as the first step toward the numerical prediction of OP, the cumulative OP (OP_tm_*) based on the top five key transition metals, namely, Cu, Mn, Fe, V, and Ni, was defined. The solubilities of metals were assumed constant over time and space based on measurements. Then, the feasibility of its prediction was verified by comparing OP_tm_* values based on simulated metals to that based on observed metals in East Asia. PM_2.5_ typically consists of primary and secondary species, while OP_tm_* only represents primary species. This disparity caused differences in the domestic contributions of PM_2.5_ and OP_tm_*, especially in large cities in western Japan. The annual mean domestic contributions of PM_2.5_ were 40%, while those of OP_tm_* ranged from 50 to 55%. Sector contributions to the OP_tm_* emissions in Japan were also assessed. The main important sectors were the road brake and iron–steel industry sectors, followed by power plants, road exhaust, and railways.

## Introduction

The aerosol oxidative potential (OP), the potential to generate reactive oxygen species (ROS) in cells that induce airway oxidative stress and inflammation, is considered to better represent the health hazards of aerosols than the mass concentration of very fine particulate matter (PM_2.5_)^1–3^. Several methods have been proposed to quantify OP^4^, and among them, the dithiothreitol (DTT) assay^5^ has been widely applied. The DTT activity is quantified as the consumption rate of the reducing agent, i.e., DTT, in buffer with the extraction of aerosols^5^. The DTT activity exhibits a stronger association than does PM_2.5_ with the fractional exhaled nitric oxide, a biomarker of airway inflammation^6^. The DTT activity has also been found to be more strongly associated with emergency department (ED) visits related to asthma/wheezing and congestive heart failure than the PM_2.5_ mass concentration^7^. Population-level analysis of the health effect of the measured ambient DTT level has demonstrated that the 3-d moving average of the DTT activity is highly associated with ED visits for multiple cardiorespiratory outcomes, especially in regard to ischemic heart disease^8^.

Even though the DTT assay is an in vitro system, the relative contributions of chemical compounds to the total DTT activity are not yet fully understood. Charrier and Anastasio^9^ indicated that water–soluble transition metals, such as Cu(II), Mn(II), Fe(II), and Fe(III), account for 80% of the DTT activity measured in California, and organics, such as phenanthrenequinone, account for 20% of the measured DTT activity. However, Nishita-Hara et al.^10^ reported, based on samples measured in Japan, that water-soluble transition metals only explain 37% and 60% of the DTT activities of fine- and coarse-mode particles, respectively. Saffari et al.^11^ demonstrated that the DTT activity is strongly associated with water-soluble and water-insoluble organics and elemental carbon. In fact, dissolved oxygen causes interfacial catalytic oxidation of DTT in the presence of elemental carbon particles^12^. Verma et al.^13^ revealed the importance of humic-like substances (HULIS), such as quinones and secondary organic aerosols, and Yu et al.^14^ indicated that the interactions between HULIS and transition metals likely contribute to the DTT activity. In addition to catalytic redox reactions of transition metals and quinones, noncatalytic DTT-active organics such as organic hydroperoxides and electron-deficient alkenes have been highlighted^15^. Thus far, the relative importance of chemical compounds to the total DTT activity is not fully understood, but the importance of the coexistence of metals and organics is widely accepted^1–3,9,14,16–18^.

To date, several experimental studies have been performed to relate OP, chemical compounds, and health outcomes^4–18^. In terms of numerical simulations, a model has been proposed to determine the chemical reactions producing ROS in epithelial lining fluids^19^ and a statistical model, called the land use regression model^20,21^, to predict the spatial variations in OP. However, none of these studies has derived the spatiotemporal variations in OP via the direct simulation using 3-dimensional numerical modeling. Very recently, Daellenbach et al.^22^ derived the spatiotemporal variations in OP in Europe by combining the 3-dimensional simulations of organic aerosols and NO_x_ and the statistical relationship between the measured OP and aerosol components in Switzerland and Liechtenstein. Daellenbach et al.^22^ did not directly predict the redox active aerosol components such as transition metals and quinones but demonstrate that the simulated OP agreed very well with that observed in the measurement sites.

Within the context mentioned above, we developed a 3-dimensional model and emission inventories of the DTT-active transition metals in Asia (TMI-Asia) and Japan (TMI-Japan) and evaluated simulation results based on field measurements in Japan^23^. Before directly predicting the total OP, as a first step, we defined the cumulative OP based only on transition metals (OP_tm_*) and assessed its predictability in this study. OP_tm_* was defined as the summation of the (simulated or observed) DTT-active transition metal concentrations multiplied by the DTT consumption rate per unit mass (obtained by laboratory experiments). This study is the first trial to directly predict the oxidative potential, but it should be noted that the contributions of other components, such as the effects of organics^22^ and the interactions between organics and metals, have not yet been considered. Many studies focused on OP only of PM_2.5_^1–9,11–21^ and so another feature of the current study is that we include the OP_tm_* contributions of coarse-mode particles and Asian dust because lung deposition of fine particulate matter (PM_10_) may be nonnegligible in some human conditions^24^, and Asian dust particles containing metals may adversely affect health^25^. Nishita-Hara et al.^10^ and Daellenbach et al.^22^ also considered the coarse-mode particles.

The main objectives of this study are thus summarized as follows: (1) to assess the predictability of OP_tm_* by numerical simulations, (2) to show the differences of horizontal distributions and the source-receptor relationship between OP_tm_* and PM_2.5_, and (3) to identify the major emission sectors for anthropogenic OP_tm_*.

## Results

### Temporal variations in OP_tm_* based on simulations and observations and their comparison

**Figure 1:**
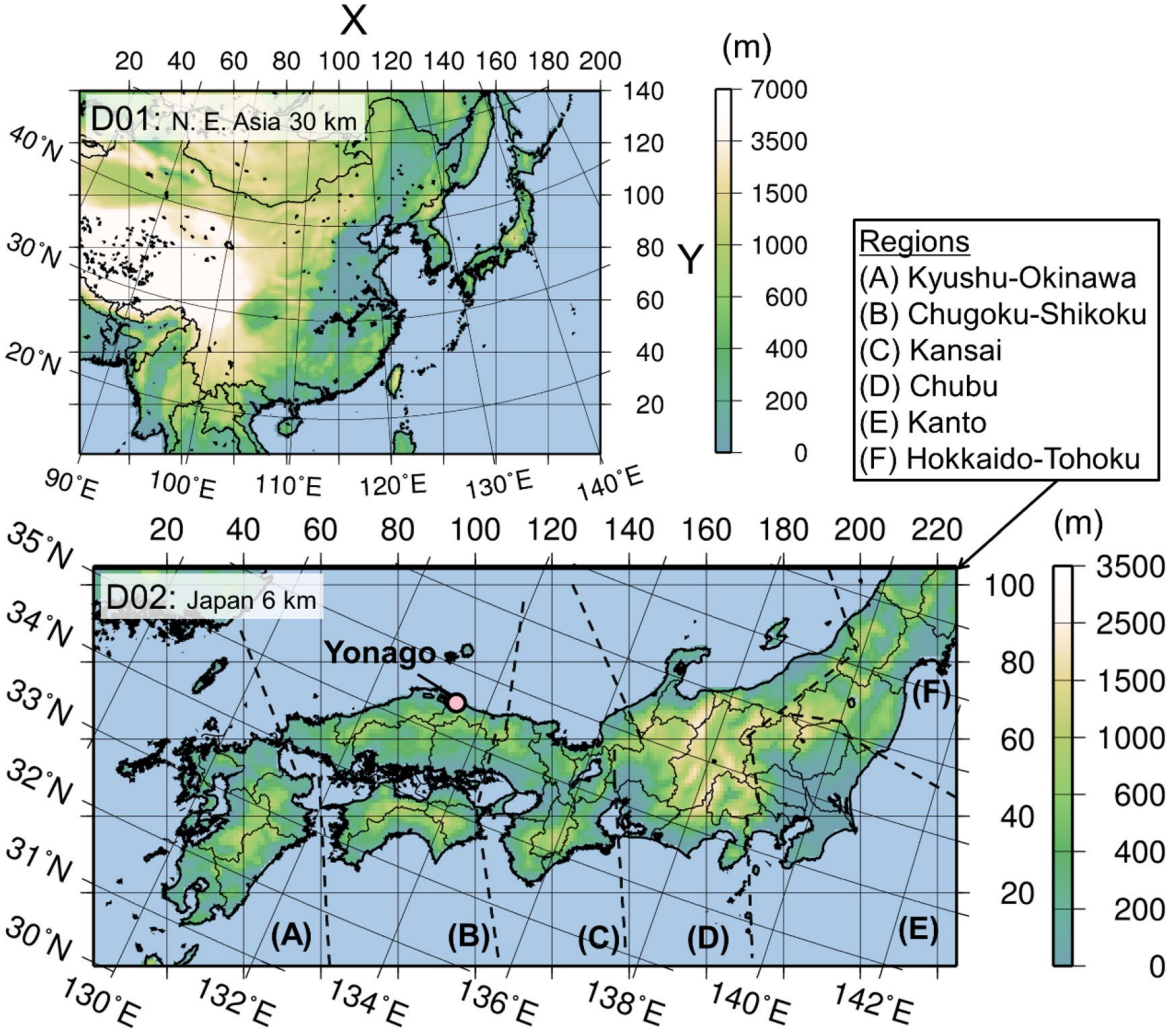
Model domains, topography, and regions considered for the analysis. The mother domain (D01) covers East Asia with Δ*x* = 30 km, and the nested domain (D02) covers the central parts of Japan with Δ*x* = 6 km. The observation site (Yonago) and regional names adopted in the analysis are shown. The national and prefecture borders are depicted in D01 and D02, respectively. This figure is identical to Fig. 1 of Kajino et al.^23^. The map was generated using the Generic Mapping Tools v4.5.7 (https://www.generic-mapping-tools.org).

Figure 1 (identical to Fig. 1 of Kajino et al.^23^) shows the mother domain (D01) and nested domain (D02) for the simulation of the transition metals and cumulative OP (OP_tm_*) based on the top five key transition metals, namely, Cu, Mn, Fe, V, and Ni, as described in Eq. (2) and Table 2 in Method section. D01 covers East Asia with a horizontal grid resolution of 30 km to simulate the long-range transport from the Asian continent to Japan via synoptic-scale disturbances such as the fronts of cyclones and migrating anticyclones. D02 covers the densely populated and industrial areas of Japan with a complex topography, and a fine grid resolution (i.e., 6 km) is necessary to accurately predict the domestic and transboundary contributions to the surface concentrations of air pollutants.

OP_tm_* based on the simulated transition metals (referred to as the simulated OP_tm_* hereinafter for simplicity) was compared to OP_tm_* based on the observed transition metals (hereinafter referred to as the observed OP_tm_*) of the total suspended particles (TSP) collected at the Yonago site (Fig. 1). As described below, the *r*_*i*_ values of Eq. (2) substantially varied among laboratories, as experimental methods such as buffer usage, pH, reaction time, and control volume differed^2^. To consider the above experimental variability, we derived two different OP_tm_* values based on *r*_*i*_ retrieved from two different experiments, namely, Charrier and Anastasio^9^ and Fujitani et al.^17^, which are referred to as OP_tm_*(CA) and OP_tm_*(F), respectively.

**Figure 2:**
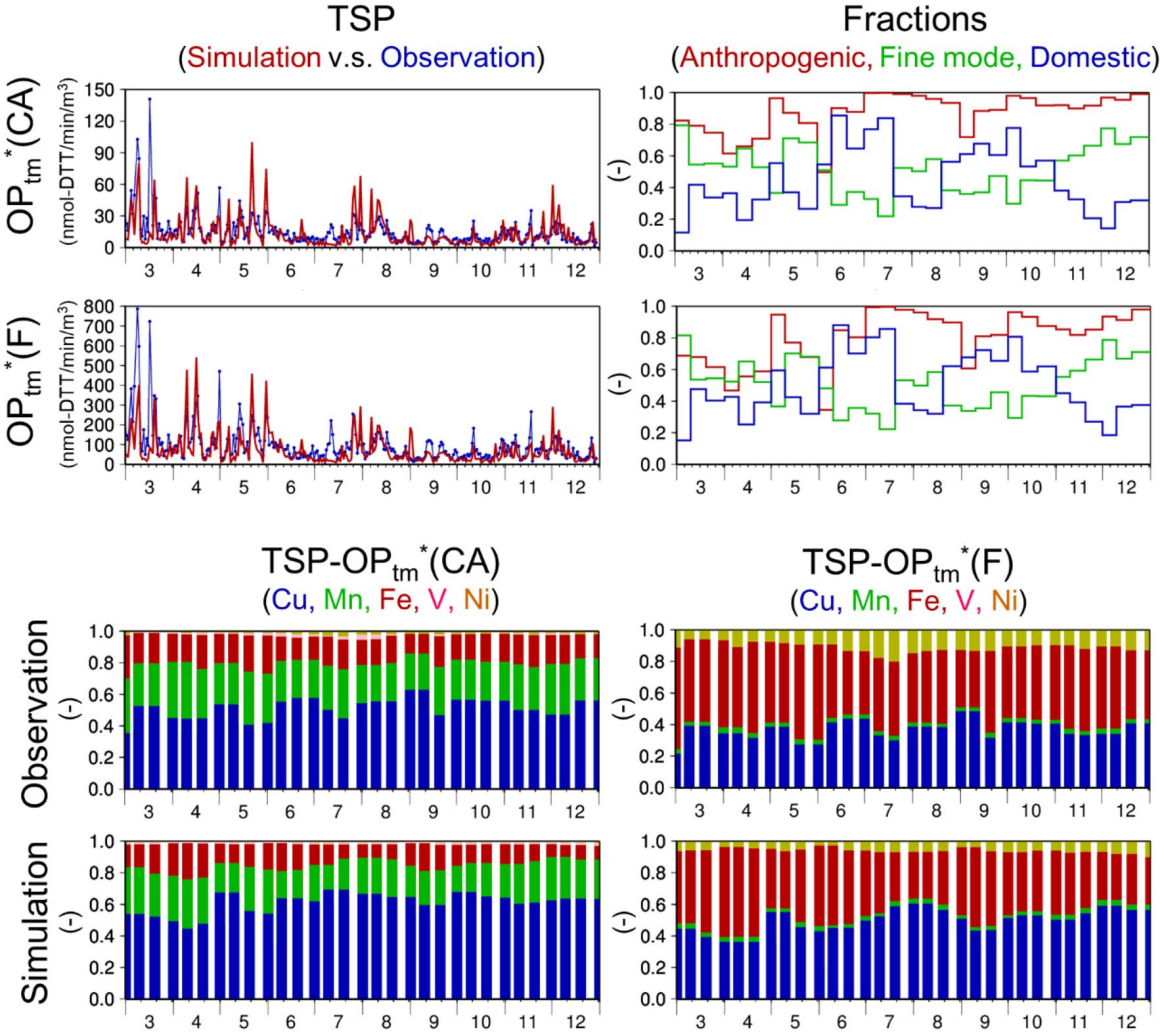
Temporal variations in the daily OP_tm_*(CA) and OP_tm_*(F) and fractions of anthropogenic vs natural compounds, fine vs coarse particles, domestic vs transboundary contributions, and DTT-active elements. Temporal variations in (upper-left panels) the daily OP_tm_*(CA) and OP_tm_*(F) based on (red) the simulated (D01) and (blue) observed transition metals in TSP (nmol-DTT min^−1^ m^−3^), (upper-right panels) 10-d mean simulated (D02) fractions of (red) anthropogenic compounds to the total compounds, (green) anthropogenic fine-mode particles to anthropogenic total particles, and (blue) anthropogenic domestic contributions to the total OP_tm_*(CA) and OP_tm_*(F), (lower-left panels) relative contributions of each metal to the observed and simulated OP_tm_*(CA) and (lower-right panels) same as the lower-left panels but for OP_tm_*(F) at Yonago. Note that the simulated concentration of TSP is equivalent to the simulated PM_10_ concentration.

Figure 2 shows a time series of the simulated (PM_10_) and observed (TSP) OP_tm_*(CA) and OP_tm_*(F) at Yonago, as well as the fractions of anthropogenic, fine-mode, and domestic components and the contributions of various elements to the simulated OP_tm_*. Statistical metrics for the comparison are summarized in Table 1. Note that the OP_tm_* simulated with Eq. (2) includes correction factor *f*_*i*_ based on measurements (the nationwide PM_2.5_ survey conducted by the Ministry of Environment, Japan (MOEJ); http://www.env.go.jp/air/osen/pm/monitoring.html; last accessed: 6 November 2020), which were independent from the Yonago data.

**Table 1:** Statistical metrics to compare the simulated (D02) and observed daily OP_tm_* values at Yonago.

Unit	*N*^a^	Observation Median^b^	*Sim:Obs*^c^	*R*^d^	*Fa2*^e^	*Fa5f.*
	–	nmol-DTT min^−1^ m^−3^	–	–	–	–
OP_tm_*(CA)	298	11.4	0.71	0.57	0.68	0.97
OP_tm_*(F)	298	93.3	0.84	0.63	0.65	0.95

^a^Number of available data.

^b^Median of the observation data.

^c^Simulation to observation median ratio.

^d^Correlation coefficient.

^e^Fraction of the simulated values within a factor of two of the observed values.

^f^Fraction of the simulated values within a factor of five of the observed values.

As indicated in Table 1, OP_tm_* was suitably predicted by the numerical simulations with correction factors based on independent MOEJ nationwide observations, even though OP_tm_* was primarily contributed by Cu and the discrepancies between the simulated and observed Cu were large (Tables 4 and 5 of Kajino et al.^23^). Nevertheless, it is not surprising because the approach was analogous to the application of a multimodel ensemble, which generally reduces the uncertainty in each model. The summation of Eq. (2) reduced the uncertainty in the simulation of each metal element. In fact, the normalized root mean square errors (NRMSE; RMSE divided by observation average) for OP_tm_*(CA) and OP_tm_*(F) in TSP at Yonago (0.47 and 0.48) were smaller than those for Cu (4.7), Fe (1.0), Mn (0.87), Ni (1.4), and V (1.4). The median values of OP_tm_*(CA) were almost one order of magnitude smaller than those of OP_tm_*(F) due to the experimental variations and thus the discussion on the absolute values of OP_tm_* is not a scope of this study. Therefore, the relative magnitudes in time and space (i.e., the temporal and spatial variations, respectively) are mainly discussed. The simulated relative contributions of metals to OP_tm_* were consistent with those based on the observations, while those to OP_tm_*(CA) and OP_tm_* (F) differed. OP_tm_*(CA) primarily consisted of Cu, followed by Mn and Fe, which was similar to measurements obtained in California. However, due to the relatively high *r*_*i*_ values for Ni and Fe(II) and relatively low *r*_*i*_ value for Mn, the major contributors of OP_tm_*(F) were Fe and Cu, followed by Ni. Although the relative contributions of each metal were different between the two methods, the relative magnitudes of anthropogenic compounds vs. Asian dust, anthropogenic fine-mode vs. coarse-mode particles, and anthropogenic domestic vs. transboundary OP_tm_* values were similar. Their variations were consistent with those in the metals, as shown in Fig. 6 of Kajino et al.^23^. Specifically, the contribution of Asian dust was large in spring, and the transboundary contribution was large in colder seasons except from late July to early August, while the fine-mode fractions were inversely correlated with the domestic contributions, which are explained in the next subsection.

### Spatial distribution and seasonal variation in OP_tm_*

**Figure 3:**
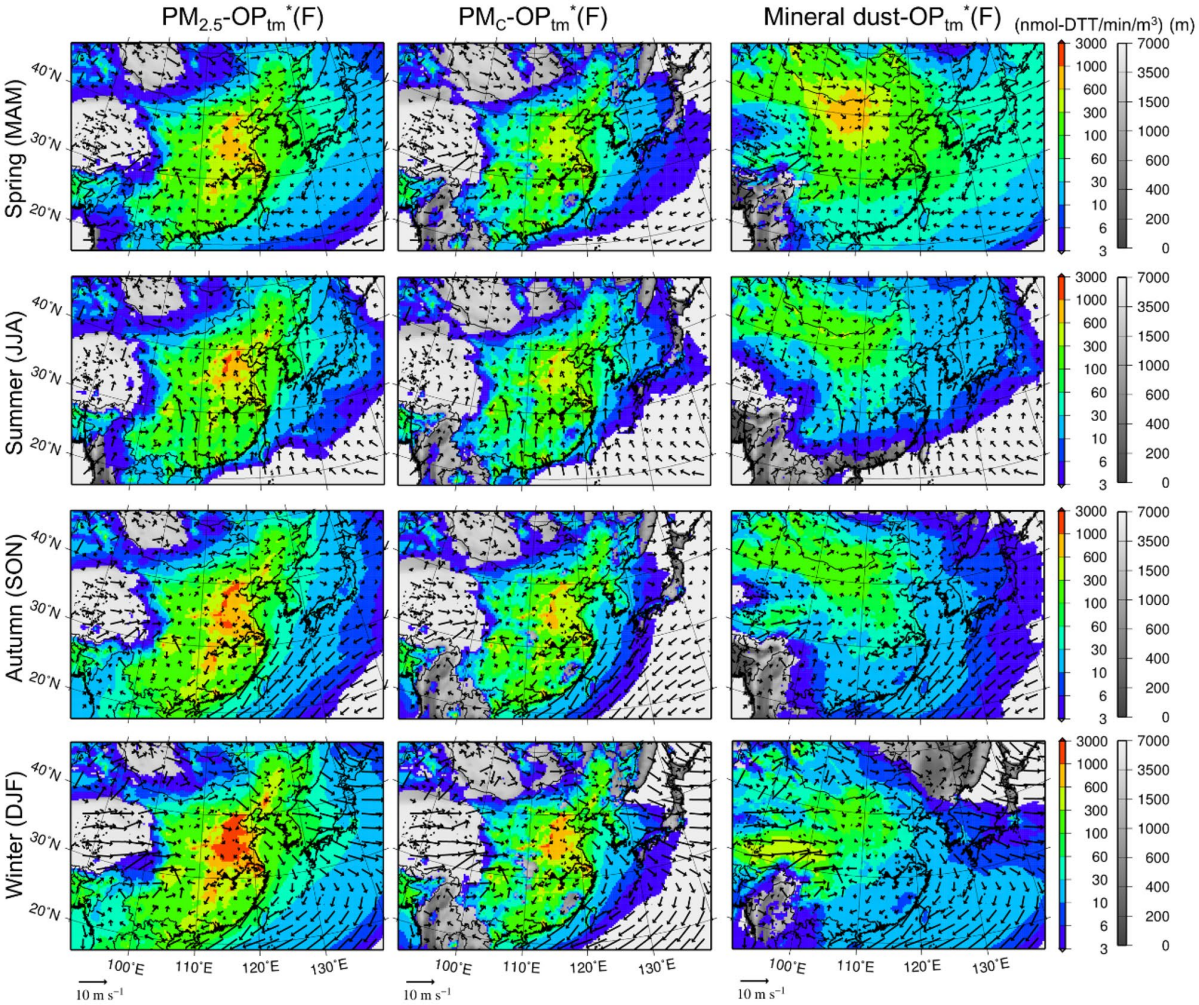
Seasonal mean surface OP_tm_*(F). Seasonal mean surface air concentrations of (left to right) anthropogenic PM_2.5_-OP_tm_*(F), anthropogenic PM_c_-OP_tm_*(F), and OP_tm_*(F) of Asian dust in (top to bottom) the spring, summer, autumn, and winter of 2013 with the surface wind vectors over D01. The model terrestrial elevations are depicted in grayscale under the various colors. The map was generated using the Generic Mapping Tools v4.5.7 (https://www.generic-mapping-tools.org).

Figure 3 shows the seasonal mean surface air concentrations of the anthropogenic PM_2.5_-OP_tm_*(F), anthropogenic coarse-mode PM_c_-OP_tm_*(F) (simulated PM_10_ minus PM_2.5_), and OP_tm_*(F) of Asian dust. The simulated OP_tm_*(CA) is not shown because the horizontal distributions were very similar.

Generally, PM_2.5_-OP_tm_*(F) is higher than PM_c_-OP_tm_*(F). These anthropogenic surface concentrations were the highest in the winter under stable meteorological conditions. However, due to the presence of surface snow, the emissions of Asian dust were suppressed in the winter. The surface concentrations of Asian dust were the highest in the spring over the Gobi Desert and were almost equivalent to those of PM_2.5_-OP_tm_*(F). As shown in Fig. 2, the long-range transport of PM_2.5_ was more prominent than that of PM_c_.

The long-range transport of air pollutants from the Asian continent to Japan is influential during the cold seasons such as the spring and winter. A westerly wind prevails in the winter, while northerly and westerly winds prevail in the spring, which results in high concentrations in the respective downwind areas. The long-range transport of Asian dust was influential in the spring. Due to the presence of a Pacific high, the long-range transport was generally insignificant in the summer. The summer of 2013 was an exception. An anticyclone persisted over the southwestern part of the Japanese archipelago from late July to mid-August, which continuously carried pollutants from the Asian continent to Japan via the marginal flow along the northern edge of the anticyclone. The seasonal mean wind pattern exhibited features of the Pacific high, but the high-surface concentration areas extended east off the coast of the continent. In fact, the domestic contribution was small during this period, as shown in Fig. 2.

### Domestic contributions of OP_tm_* and differences from those of PM_2.5_

**Figure 4:**
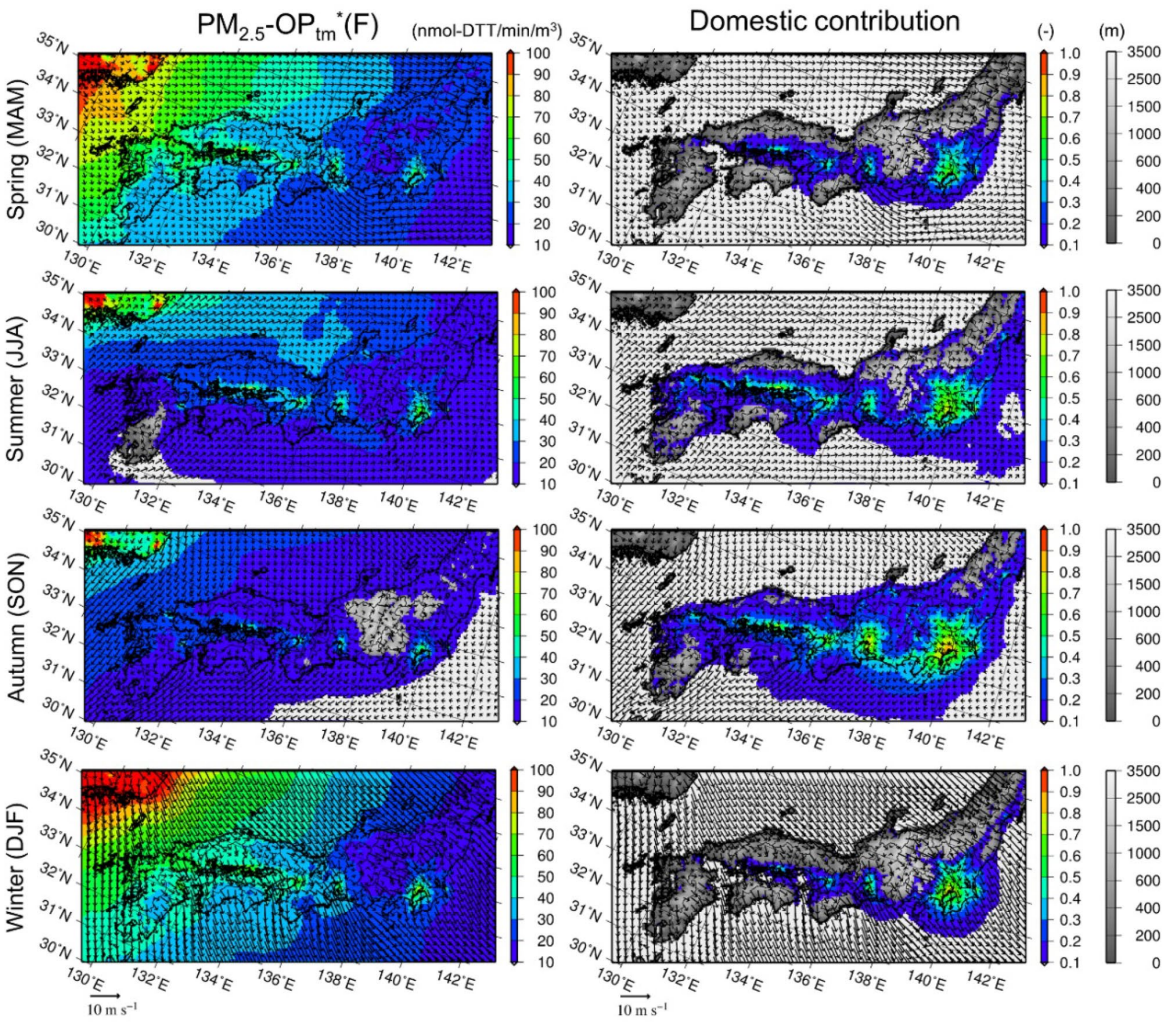
Seasonal mean surface anthropogenic PM_2.5_-OP_tm_*(F) and its domestic contributions. Seasonal mean (left) surface air concentrations of anthropogenic PM_2.5_-OP_tm_*(F) (nmol-DTT min^−1^ m^−3^) and (right) its domestic contributions over D02 in (top to bottom) the spring, summer, autumn, and winter of 2013. The model terrestrial elevations are depicted in grayscale under the various colors. The map was generated using the Generic Mapping Tools v4.5.7 (https://www.generic-mapping-tools.org).

The spatial distribution of the simulated anthropogenic OP_tm_*(F) in PM_2.5_ over D02, together with its domestic contributions, is shown in Fig. 4. A contrast between the domestic and transboundary components and their seasonal differences are clearly observed in the figure. Transboundary air pollution dominated in the spring and winter, and there was a clear horizontal gradient in the surface PM_2.5_-OP_tm_*(F) concentrations from west to east during that season. However, high surface PM_2.5_-OP_tm_*(F) concentrations were found in Kanto, including the Tokyo Metropolitan Area, and the domestic contribution exceeded 50% throughout the year. In addition to the Kanto region, high-concentration areas were observed around large cities, such as Nagoya (in Chubu) and Osaka (in Kansai), where the domestic contributions were as large as those in Kanto (even though the areas were smaller). The domestic contributions were large over the inland seas and their surroundings in the western part of Japan, such as the Seto Inland Sea between Chugoku, Shikoku, and Kyushu and the Bungo Channel between Kyushu and Shikoku. Under the strong influence of the transboundary transport in the spring and winter, the concentrations over the areas were higher than those in the other areas at the same longitudes. The Seto Inland Sea is a major route of vessels in Japan, and thus, large industrial regions are located along the coast, and as a result, the transition metal emissions from ships and industries are high in this region, as shown in Fig. 6.

**Figure 5:**
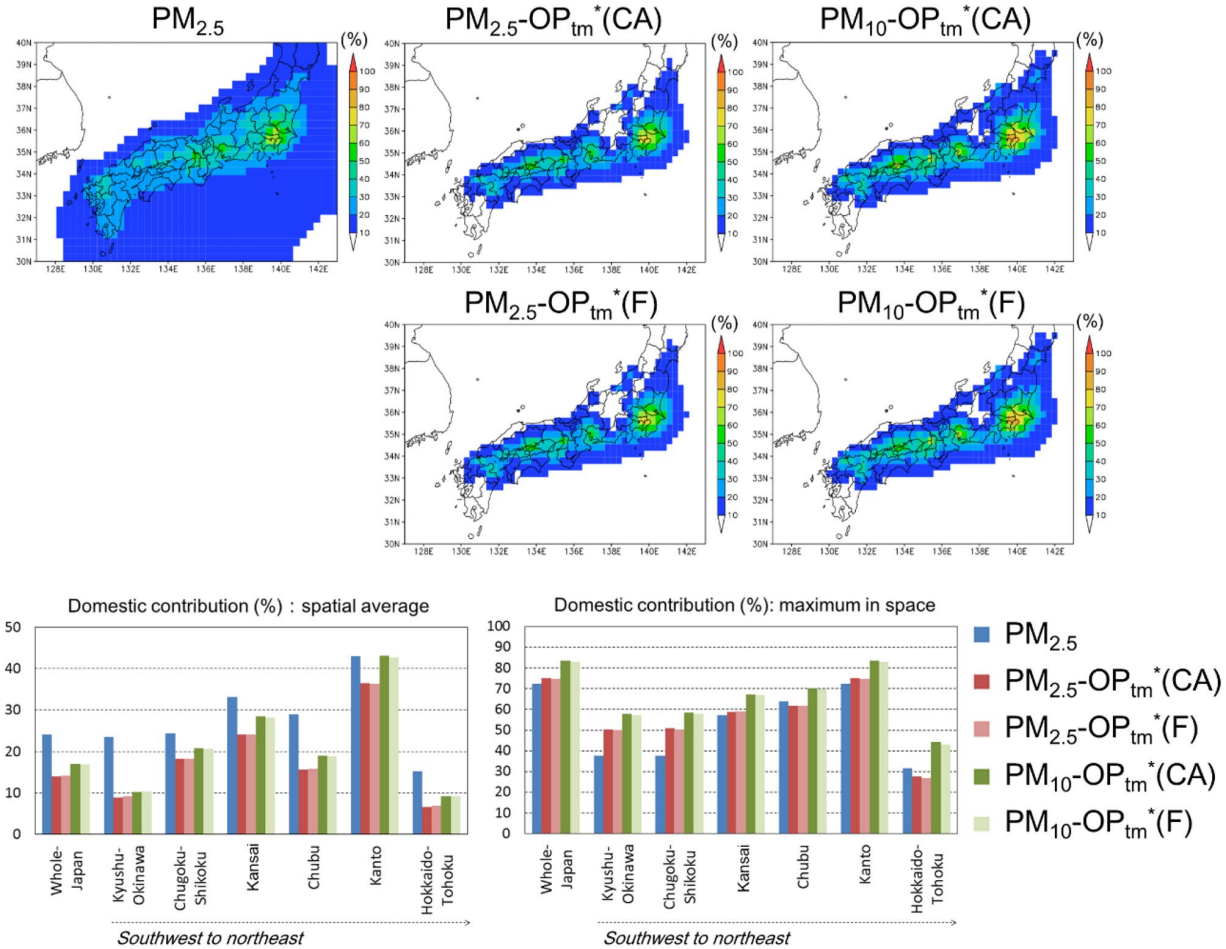
Annual mean domestic contributions of PM_2.5_ and OP_tm_*(F). (Top five panels) The annual mean domestic contributions of PM_2.5_ simulated by Kajino^26^ and anthropogenic OP_tm_*(CA) and OP_tm_*(F) in PM_2.5_ and PM_10_ simulated in this study. (Bottom two panels) (%) (left) spatially averaged values and (right) maximum values in space of the domestic contributions over all of Japan and the six regions, as shown in Fig. 1. The map was generated using the Grid Analysis and Display System (GrADS) v2.0.2, available at http://cola.gmu.edu/grads.

To determine the differences between the health hazard based on OP and the conventional health hazard, i.e., the PM_2.5_ mass concentration, the domestic contributions of PM_2.5_ and OP_tm_* are shown and compared in Fig. 5. The simulation results of PM_2.5_ were retrieved from a previous study^26^ using the same bottom-up inventory^27^ and the same simulation period (2013) with a domain similar to D01 at a different grid resolution (36 km). To quantitatively compare the OP_tm_* simulation results to the simulated PM_2.5_ concentrations (using a common inventory and similar resolution) and to estimate the quantities in the regions outside of D02 (such as Hokkaido, all of Tohoku, and Okinawa), the simulation results based on D01 were considered for the comparison.

The horizontal distributions of the domestic contributions of OP_tm_*(CA) and OP_tm_*(F) were very similar. The distributions of the domestic contributions of PM_2.5_ were much broader than those of the domestic contributions of OP_tm_*. This result occurred due to the difference in the contributions of the secondary aerosols. OP_tm_* is composed only of primary aerosols, i.e., metal elements, while PM_2.5_ is composed of both primary and secondary aerosols. Generally, the relative contributions of secondary aerosols are larger in downwind regions (after long-range transport). Consequently, the domestic contribution of OP_tm_* is the largest near the source regions, while that of PM_2.5_ is larger in the downwind regions. As a result, the areal mean values of the PM_2.5_ domestic contributions are larger than those of PM_2.5_-OP_tm_*, but the areal maximum values of PM_2.5_-OP_tm_* are as large or even significantly larger than those of PM_2.5_ especially over the Kyushu-Okinawa and Chugoku-Shikoku regions, where long-range transport is predominant. In these regions, more than 60% of PM_2.5_ was attributed to transboundary contributions and 40% was attributed to domestic contributions. However, in regard to OP, the domestic contribution was as high as 50%. The domestic contributions of PM_10_-OP_tm_* were generally larger than those of PM_2.5_-PM_tm_* by up to 5% in terms of the areal average or approximately 10% in terms of the areal maximum. As previously mentioned, this result occurred because the lifetime of PM_10_ is generally shorter than that of PM_2.5_. Hence, the relative contributions of long-range transported PM_10_ are lower than those of PM_2.5_.

### Contributions of the emission sectors to OP_tm_* in Japan

**Figure 6:**
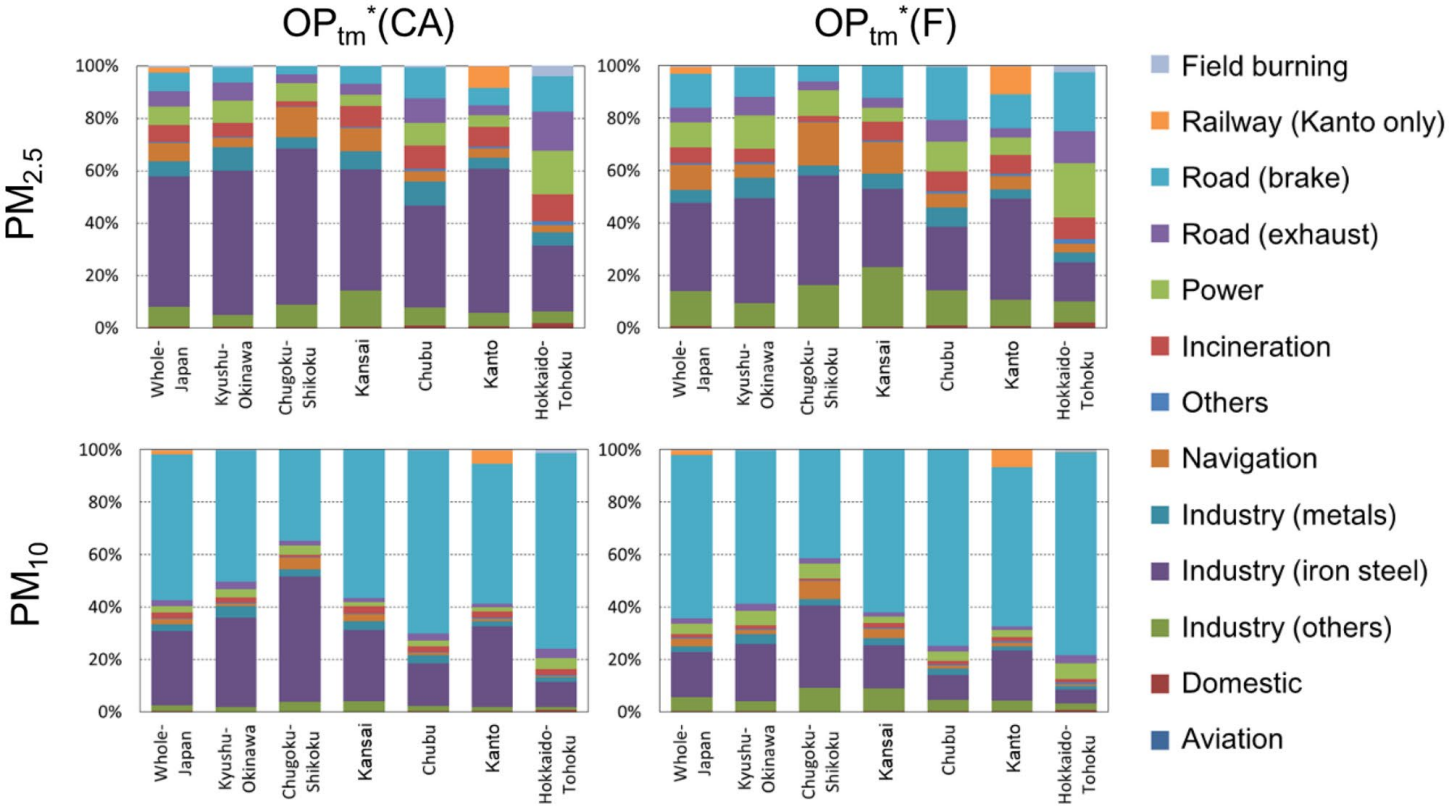
Relative contributions of the emission sectors to OP_tm_*. Contributions of each anthropogenic emission sector in TMI-Japan (v1.0) to OP_tm_*(CA) and OP_tm_*(F) in regard to PM_2.5_ and PM_10_ over all of Japan and the six regions, as shown in Fig. 1. Note that TMI-Japan (v1.0) provides railway emissions only for Kanto.

The relative contributions of each emission sector to OP_tm_*(CA) and OP_tm_*(F) of PM_2.5_ and PM_10_ are shown in Fig. 6. While large differences occur in the metal contributions to OP_tm_*(CA) and OP_tm_*(F), the sector contributions based on these two methods are not very different both in terms of PM_2.5_ and PM_10_. However, the sector contributions to PM_2.5_ and PM_10_-OP_tm_* are very different. For example, the road brake sector is the top contributor to PM_10_-OP_tm_* but not to PM_2.5_-OP_tm_*. It should be noted that the size distribution of the current inventory has not yet been evaluated. In fact, a recent laboratory experiment^28^ has demonstrated that most brake wear particles occur in the fine mode, i.e., PM_2.5_. The size apportionment of the emission inventory certainly requires further improvement. The results provided by TMI-Japan are presented below in this section.

The most important sector in regard to PM_10_-OP_tm_* was the road brake sector, followed by the iron-steel industry sector. Regarding PM_2.5_-OP_tm_*, the other sectors such as other industries (nonmetals), navigation, incineration, power plants, road exhaust, and railways attained almost equal contributions (ranging from only a few percent up to 10–20%). As shown in Fig. 2, the source contributions mainly reflected those of Cu and Fe followed by those of Mn and Ni. The large contribution of the road brake sector to PM_10_-OP_tm_* originated from Cu and Fe and that to PM_2.5_ originated from Fe. The iron-steel industry contribution to OP_tm_* originated primarily from Fe and Mn, while it originated from Ni and Cu in regard to PM_2.5_. The contribution of the other industry (nonmetals) sector to PM_2.5_-OP_tm_* originated from Ni and Cu, and the contributions of the metal industry sectors other than the iron-steel and incineration sectors originated from Cu and Fe. The power plant sector emitted Fe and Cu, which contributed to PM_2.5_-OP_tm_*. The contribution of the road exhaust sector to PM_2.5_-OP_tm_*(F) mostly originated from Cu.

The contribution of the navigation sector was the largest in Chugoku-Shikoku, originating from V and Ni. The contribution of the navigation sector was large in regard to PM_2.5_-OP_tm_*(F) due to the high *r*_*i*_ value for Ni. Vanadium (V) and Ni achieved almost equal contributions to PM_2.5_-OP_tm_*(F). The metal emission amounts were the largest in Kanto, the most populated region of Japan, in terms of Cu, Mn, and Fe, while the V and Ni emissions were the largest in Chugoku-Shikoku. As previously described, this result occurred due to the aerosols generated during heavy fuel oil combustion emitted from vessels in the Seto Inland Sea and industrial factories along the coast.

The railway sector contributed approximately 10% to PM_2.5_-OP_tm_* in Kanto, which was primarily attributed to Fe (Fe stemming from the railway sector accounted for approximately 15% of the total Fe emission) and Cu. This sector also emitted Mn. Because railway emission data were available only for Kanto, the OP_tm_* and metal emissions in the other regions could be underestimated at similar fractions.

## Discussion

### Toward the effective emission control measures

Daellenbach et al.^22^ and this study indicated that the emission sources for PM_2.5_ and OP could be very different. There may be a possibility that an emission control measure to reduce PM_2.5_ surface air concentrations may not necessarily reduce their health risk, if OP is essential to the health hazard of aerosols and if the selected control measure does not reduce OP. For example, reduction of ammonia emission may lead to decrease in surface air concentrations of PM_2.5_, but not OP because the emission sources of transition metals and quinones are different from those of ammonia. Besides, decrease of ammonia in the air can lead to increase of aerosol acidity and thus solubility of metals, which could enhance OP of aerosols. On the other hand, even though an emission control measure does not significantly decrease the PM_2.5_ concentration, it may be effective if it decreases OP efficiently.

Certainly, OP is not a sole and perfect health hazard of aerosols, but concerning only PM_2.5_ may mislead the emission control strategy. In order to seek for the better and effective control measures, OP must be directly simulated by numerical models in addition to PM_2.5_ and other conventional aerosol components, because numerical models are one of the most powerful tools to quantitatively evaluate the impacts of emission control on the surface air concentrations.

### Future research

The current study has the following limitations, which should be resolved in the future. We only simulated the total (water-soluble and water-insoluble) metal concentrations and assumed a constant solubility over time and space. Water solubility of metals depends on their chemical forms and aerosol acidity. As acidity is higher, some of transition metals such as Cu, Mn, and Fe become more water soluble. We should consider specific water solubilities of metals for each emission source and simulate any changes in water solubility of metals due to changes in aerosol acidity occurring during transport^29^. We only considered metals but organics, especially quinones, are important DTT-active agents^9,11–13^. The interactions between metals and organics can also enhance DTT consumption and thus should be considered^14,19^. Metals are primary species, but quinones are secondary and produced in the air. During transport from emission source to downwind regions, the relative contributions of quinones to OP may increase, which can cause differences in dominant emission sectors affecting OP in the two regions. There are species that are not redox active but cause a notable oxidative stress, such as endotoxin^30^. These species should also be taken into account in the simulation. Finally, epidemiological studies are required to assess the applicability of the new health hazards^6–8,31^. Further integration of meteorology, chemistry, toxicology, and epidemiology is indispensable in the next study stages.

## Methods

### Numerical simulations of the transition metals

A transition metal version^23^ of the Japan Meteorological Agency (JMA) regional-scale meteorology-chemistry model (NHM-Chem^32^) was adopted in this study. We retrieved simulation results of the considered transition metals from Kajino et al.^23^, and details are not presented here.

The transition metal version of NHM-Chem employs three aerosol categories: anthropogenic submicron particles (SUB), anthropogenic coarse-mode particles (COR) and mineral dust (MD). The full chemistry version of NHM-Chem fully considers aerosol microphysical processes such as nucleation, condensation, coagulation, and deposition, but the transition metal version only considers deposition processes, such as wet deposition (in-cloud and below-cloud deposition), fog deposition (contact of cloud droplets in the bottom layers of the model to the ground surface^33^), and dry deposition. Upon emission, the size distributions of the above three aerosol categories are prescribed^23^, which change during transport only due to advection, turbulent diffusion, and removal processes. A prescribed hygroscopicity is assumed for each category^23^, which was adopted to obtain cloud condensation nuclei activity used for in-cloud scavenging and fog deposition and hygroscopic growth used for below-cloud scavenging and dry deposition.

As shown in Fig. 1, two model domains were applied to simulate the long-range transport from the Asian continent to Japan and the contributions of local emissions and local transport in Japan. D01 covers East Asian countries with a grid spacing (Δ*x*) of 30 km and 200 × 140 grid cells on the Lambert conformal conic projection to resolve the transport of air pollutants due to synoptic-scale disturbances. D02 covers the Kyushu, Shikoku, and Honshu (only the Chugoku, Kansai, Chubu, and Kanto regions and part of the Tohoku region) islands of Japan with Δ*x* = 6 km and 226 × 106 grid cells on the Lambert conformal conic projection. The horizontal grids of the meteorological and transport parts of NHM-Chem are the same, but the vertical grids differ. There are 38 vertical levels up to 22,055 m above sea level (a.s.l.) for the meteorological simulations and 40 levels up to 18,000 m a.s.l. for the transport simulations. A JRA-55 global analysis^34^ (1.25° × 1.25°, 6 h) was applied to the initial and boundary conditions of the meteorological simulations in D01. The JMA meso-regional objective analysis (MANAL; 5 km × 5 km, 3 h) was adopted in D02. These analysis data were also used for the spectral nudging of the meteorological simulations.

Kajino et al.^23^ developed emission inventories of eight DTT-active metals, namely, Cu, Mn, V, Ni, Pb, Fe, Zn, and Cr, in anthropogenic PM_2.5_ and PM_10_ in Asia and Japan, referred to as Transition Metal Inventory (TMI)-Asia v1.0 (Δ*x* = 0.25°; monthly, 2000–2008; 9 sectors) and TMI-Japan v1.0 (Δ*x* = 2 km; hourly, weekday/weekend; monthly, 2010; 29 sectors), respectively. Kajino et al.^23^ also simulated metals originating from Asian dust. TMI-Asia and TMI-Japan were used for the transport simulations over D01 and D02, respectively. Anthropogenic PM_2.5_ and PM_10_ emissions were allocated to the SUB and COR categories, and those originating from Asian dust were allocated to the MD category. The simulated metal concentrations of SUB were compared to MOEJ PM_2.5_ concentration measurements, and the simulated concentrations of COR plus MD were compared to measurements of TSP reported in Kajino et al.^23^ and this study. A part of mineral dust mass should be included in PM_2.5_ in reality, but it was neglected in this study.

### Surface air concentration measurements of the transition metals

To derive the observed OP_tm_*, we used the same observation datasets as that reported in Kajino et al.^23^. The measurement data were collected in Yonago city, Tottori Prefecture, Japan (Fig. 1), from March to December 2013. Aerosols were collected with a TSP sampler (MCAS-03, Murata Keisokuki Service Co. Ltd.) on polytetrafluoroethylene (PTFE) filters (Whatman, PM_2.5_ Air Monitoring PTFE Membrane Filter, 46.2 mm φ) at a flow rate of 30 L min^−1^. The sampler was situated on a rooftop terrace of the building of the Faculty of Medicine, Tottori University (35.43°N, 133.33°E), approximately 20 m above ground level. The inorganic elements were analyzed using the fundamental parameter quantification method of energy-dispersive X-ray fluorescence spectrometry (EDXRF-FP), which was developed and evaluated by Okuda et al.^35,36^.

### Definition and derivation of the cumulative oxidative potential based on transition metals (OP_tm_*)

In this section, we assessed the model predictability of the observed cumulative OP based on the top five DTT-consuming metals in air determined via reagent experiments. It should be noted that this parameter is not the realistic OP in the atmosphere but the idealized OP. The realistic OP can be expressed as follows:

1 $${\text{OP}} = \sum\limits_{i} {r_{i} \times C_{i} } + \sum\limits_{j} {r_{j} } \times C_{j} + \sum\limits_{i} {\sum\limits_{j} {f_{i,j} \left( {C_{i} ,C_{j} } \right)} } ,$$

where *r*_*i*_ and *r*_*j*_ are the specific OP values (DTT consumption rate per unit of mass) of metals *i* and organic compounds *j*, respectively, *C*_*i*_ and *C*_*j*_ are the surface air concentrations, and *i* and *j* are the metal ions and organic compounds, respectively. The last term is an interaction term between the metal ions and organic compounds^14^.

However, because water solubility data of the metals and DTT-active organic compounds are not available in the inventory and model, we defined the cumulative OP based only on the total (soluble + insoluble) metals (OP_tm_*) as follows:

2 $${\text{OP}}_{{{\text{tm}}}}^{*} = \sum\limits_{i} {r_{i} \times } \chi_{const,i} \times T_{i} \times f_{i},$$

where *r*_*i*_, *χ*_*const,i*_, *T*_*i*_, and *f*_*i*_ are the specific OP, water solubility (assumed constant in time and space), total surface air concentration, and simulation bias correction factor, respectively (*i*: top five DTT-consuming metals, namely, Cu, Mn, Fe, V, and Ni). The values used in Eq. (2) are listed in Table 2. It is known that the *r*_*i*_ values vary among laboratories, as experimental methods such as buffer usage, pH, reaction time, and control volume are different^2^. To consider the variability in experiments, two different OP_tm_* values were derived using the *r*_*i*_ values of Charrier and Anastasio^9^ and Fujitani et al.^17^, referred to as OP_tm_*(CA) and OP_tm_*(F), respectively. Charrier and Anastasio^9^ and Fujitani et al.^17^ provided fitted *r*_*i*_ values by using exponential functions for specific species, but the *r*_*i*_ values of all species were fitted with linear functions in this study. Because there was no water solubility information available in the measurements or emission profiles, constant values of *χ*_*i*_ were assumed and applied, which were obtained from Okuda et al.^37^. Moreover, *f*_*i*_ was equal to 1 for the observed OP_tm_*, while the inverse of the *Sim:Obs* ratio obtained from the comparison of the D02 simulations and nationwide PM_2.5_ measurements of the MOEJ was adopted for *f*_*i*_ in regard to the simulated OP_tm_*. The same *f*_*i*_ was applied for PM_2.5_-OP_tm_*, PM_10_-OP_tm_*, and TSP-OP_tm_*. Although *f*_*i*_ was derived based on simulated and observed PM_2.5_ without consideration of simulated PM_2.5_ fraction of mineral dust, it was proved to be reasonable from the comparisons of simulated and observed TSP-OP_tm_* at Yonago as shown in Table 1. In this paper, OP_tm_* based on the simulated and observed transition metal concentrations was simply referred to as the simulated OP_tm_* and observed OP_tm_*, respectively.

**Table 2:** Values assumed for the parameters of Eq. (2) to estimate OP_tm_*(CA) and OP_tm_*(F).

Unit	*r*_*i*_ for OP_tm_*(CA)	*r*_*i*_ for OP_tm_*(F)	*χ*_*i*_	*f*_*i*_
	μM-DTT min^−1^ μM^−1^	μM-DTT min^−1^ μM^−1^		
Cu(II)	1.80	7.69	0.66	0.303
Mn(II)	0.720	0.398	0.54	0.833
Fe(*)^a^	0.0361	0.616	0.23	0.909
V(V)	0.101	0	0.90	0.385
Ni(II)	0.106	4.08	0.73	0.270

^a^Assuming the equal presence of Fe(II) and Fe(III).

## Data Availability

The simulated and observed data used for the figures and tables are available at https://mri-2.mri-jma.go.jp/owncloud/s/pfAEWT3Qi3EyGMY (last accessed: 2 November 2020).

## Code availability

The NHM-Chem source code is available subject to a license agreement with the JMA. Further information is available at http://www.mri-jma.go.jp/Dep/glb/nhmchem_model/application_en.html (last accessed: 2 November 2020).
